# Hypothetical Role of Multiwavelength Diode Laser (755, 808, and 1064 nm) in Addressing Bony Resorption in Facial Aging: A Clinical Commentary

**DOI:** 10.1111/jocd.70348

**Published:** 2025-07-15

**Authors:** Jovian Wan, Song Eun Yoon, Jong Keun Song, Tingsong Lim, Jin‐Hyun Kim, Soobin Kim, Kyu‐Ho Yi

**Affiliations:** ^1^ Medical Research Inc. Wonju Republic of Korea; ^2^ BRANDNEW Aesthetic Surgery Clinic Seoul Korea; ^3^ Pixelab Plastic Surgery Clinic Seoul Korea; ^4^ Clique Clinic Kuala Lumpur Malaysia; ^5^ You and I Clinic Seoul Republic of Korea; ^6^ Institute of Biomaterial Implant, Department of Oral Anatomy, College of Dentistry Wonkwang University Iksan Republic of Korea; ^7^ Division in Anatomy and Developmental Biology, Department of Oral Biology, Human Identification Research Institute, BK21 FOUR Project Yonsei University College of Dentistry Seoul Korea

**Keywords:** aesthetic medicine, aging face, diode laser, facial rejuvenation, non‐invasive

## Abstract

**Background:**

Facial aging involves not only soft tissue laxity but also progressive bony resorption, particularly in the maxilla and mandible. Current non‐invasive aesthetic modalities primarily target soft tissues, neglecting skeletal changes that contribute to volume loss and structural descent.

**Objective:**

To propose the hypothetical role of multiwavelength diode laser (MWDL) in stimulating osteogenesis via controlled thermal delivery to the periosteum, offering a novel, non‐invasive strategy for counteracting facial bone resorption.

**Methods:**

This clinical commentary discusses MWDL's mechanism—targeting the periosteal layer using 755, 808, and 1064 nm wavelengths (N.CORE 3D (previous version of Fortra), Classys Inc., Seoul) to deliver 43°C–46°C heat. A case study of a 70‐year‐old female receiving 16 sessions over 4 years was included. Cortical bone thickness was measured pre‐ and post‐treatment using dental radiographs.

**Results:**

Modest increases in cortical bone thickness were observed: from 3.4 to 3.51 mm (left) and from 3.14 to 3.37 mm (right), suggesting possible periosteal osteogenic stimulation.

**Conclusion:**

MWDL may represent a promising adjunct to soft tissue rejuvenation by addressing age‐related skeletal decline. While early findings are encouraging, controlled trials are needed to validate efficacy, optimise protocols, and define long‐term safety.

## 1 Introduction

Facial aging is a complex, multifactorial process characterized by the progressive loss of soft tissues, including the skin, fat, and muscle, alongside notable alterations in the underlying skeletal framework. One of the less commonly addressed yet crucial aspects of this process is bony resorption, which involves the gradual thinning and resorption of bone. This reduction in bone support significantly contributes to the hollow and sagging appearance frequently associated with aging [1, 2].

While many rejuvenation treatments primarily focus on enhancing the skin and soft tissues, the underlying bone structure plays a crucial role in maintaining facial integrity. Therefore, addressing bone loss is essential for achieving more comprehensive and long‐lasting rejuvenation. In this context, we explore a novel approach: heat‐stimuli‐enhanced osteogenesis using multiwavelength diode laser (MWDL) technology. This innovative treatment targets the periosteal layer, stimulating osteoblast activity to promote new bone formation. By promoting osteogenesis, MWDL holds the potential to rebuild bony support and restore facial volume, addressing a critical aspect of aging that soft tissue treatments alone cannot resolve.

## 2 Bony Resorption and Its Role in Facial Aging

Bone resorption is an inevitable part of the aging process. The facial skeleton undergoes remodeling, with notable losses in the maxilla, mandible, and zygomatic arches. These changes lead to the characteristic hollowing of the cheeks, deepened nasolabial folds, and the development of jowls. The reduction in bone volume diminishes the support for overlying soft tissues, exacerbating the sagging and drooping effects of aging [2, 3].

Traditional aesthetic interventions, such as dermal fillers and fat grafting, provide temporary solutions by augmenting soft tissue volume [4, 5]. However, these approaches do not address the underlying issue of bone resorption. As the skeletal framework continues to deteriorate, the efficacy of these treatments diminishes, often necessitating more frequent maintenance to preserve their initial effects. Without targeting the foundational bone loss, these interventions are limited in their capacity to deliver sustained and comprehensive facial rejuvenation.

## 3 Mechanism of Action: Stimulating Osteogenesis Through Heat‐Stimuli

The periosteum is a highly vascularised tissue layer covering the bones, rich in osteoprogenitor cells that can differentiate into osteoblasts. These osteoblasts are responsible for forming new bone matrix, which eventually mineralizes to become bone [6]. MWDL technology uses multiple laser wavelengths to penetrate deep into the skin, targeting the periosteum and delivering heat to activate these osteoprogenitor cells [7].

Heat stimuli within a controlled temperature range (typically 43°C–46°C) have been shown to induce osteogenesis, or bone formation, by stimulating osteoblast activity. This process mimics the body's natural response to injury, wherein thermal stress enhances blood flow and nutrient delivery to the affected area, promoting tissue regeneration [8].

Multiwavelength diode lasers operate at different wavelengths, such as 755, 808, and 1064 nm (N.CORE 3D (previous version of Fortra), Classys Inc., Seoul), allowing for precise penetration into various depths of tissue [9]. By directing heat specifically to the periosteal layer, MWDL has the potential to stimulate new bone formation without causing damage to surrounding soft tissues. This ability to target bone regeneration at the structural level offers a promising solution for addressing age‐related bone loss in the face.

## 4 Clinical Insights From Multiwavelength Diode Laser Technology

Choi and Yi [7] have highlighted the success of MWDL technology in a case series, where it was used for soft tissue tightening and facial sculpting.

Building on these findings, we hypothesize that directing the heat generated by MWDL specifically to the periosteum could more effectively activate osteoblasts and stimulate collagen synthesis, leading to enhanced bone formation. This hypothesis is grounded in the principle that heat can promote osteogenesis, and MWDL's ability to deliver controlled and targeted heat offers an opportunity to optimize this process.

MWDL technology utilizes multiple laser wavelengths, which allows for precise heating of the periosteal layer. This level of control may enable deeper penetration into bone tissue, potentially resulting in more substantial osteogenesis and bone remodeling than what is achievable with current techniques. Importantly, MWDL remains a non‐invasive approach, making it an attractive option for patients who may not be suitable candidates for invasive procedures, such as bone grafts or implants.

## 5 Potential Clinical Applications of Multiwavelength Diode Laser Technology in Facial Rejuvenation

MWDL is particularly promising for patients with moderate to severe bone loss in key areas such as the midface, jawline, and periorbital region—regions essential for maintaining facial contour and symmetry. The degeneration of bone in these areas leads to some of the most prominent signs of aging.

By stimulating new bone formation, MWDL could help restore facial structure, yielding a more youthful appearance. Unlike fillers, which provide temporary volume restoration, MWDL offers the potential for longer‐lasting results by addressing the underlying cause of volume loss—bone resorption. Moreover, MWDL could be used in conjunction with soft tissue treatments to further enhance both their effectiveness and longevity.

## 6 Mechanistic Considerations: Osteoblast Activation and Bone Remodeling

Heat stimuli have been shown to enhance osteoblast activity in both in vivo and in vitro models. Osteoblasts, the cells responsible for new bone formation, are particularly responsive to controlled thermal stress. In a study by Ota et al., rats treated with heat stimuli at temperatures between 43°C and 46°C exhibited significant increases in bone formation compared to controls. This was attributed to the activation of osteoblasts in the periosteal layer, which produced new bone matrix at the site of thermal stimulation.

Interestingly, the effects of heat on bone are not uniform across all cell types. While osteoblasts show increased activity in response to heat, chondrocytes do not exhibit the same response. This suggests that heat stimuli specifically promote osteoblastic ossification, rather than endochondral ossification, where cartilage is formed before being replaced by bone [8]. Therefore, MWDL's targeted approach is particularly suited for promoting direct bone regeneration in areas affected by bony resorption.

## 7 Safety and Efficacy of Multiwavelength Diode Laser Technology

The precision of MWDL minimizes the risk of damage to surrounding tissues, making it a safe option for patients [7, 10]. Unlike invasive procedures, MWDL does not require long recovery times or pose significant risks of infection or scarring.

However, treatment protocols must be carefully calibrated to avoid excessive heat, which could lead to osteocyte apoptosis or damage to adjacent tissues.

While initial studies have shown promising results, further research is required to establish optimal MWDL treatment parameters, including wavelength selection, energy levels, and treatment durations. Large‐scale clinical trials are needed to assess the long‐term benefits and safety of MWDL in human subjects.

## 8 Case Study

This study was conducted in accordance with the principles of the Declaration of Helsinki. The study was conducted using retrospective data of the patient. The patient provided written informed consent for both the treatment and the publication of her clinical information and images. As this was a clinical observation without experimental intervention, approval from an institutional ethics committee was not required.

To further illustrate the potential of MWDL technology in addressing bony resorption, we present a case study of a 70‐year‐old female patient who underwent MWDL treatment (N.CORE 3D (previous version of Fortra), Classys Inc., Seoul) for facial rejuvenation. Pre‐ and post‐treatment dental X‐rays were used to assess changes in cortical bone thickness, a key indicator of bone density and structural integrity.

**FIGURE 1:**
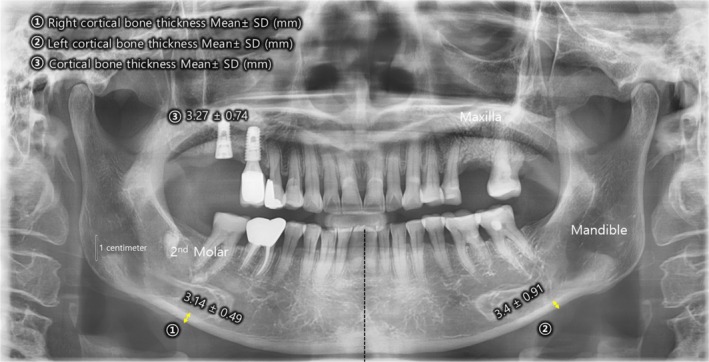
Pre‐treatment dental X‐ray of a 70‐year‐old female patient undergoing MWDL therapy for facial rejuvenation. The cortical bone thickness measured 3.4 ± 0.91 mm on the left side and 3.14 ± 0.49 mm on the right side, indicating age‐related bone loss in the maxilla and mandible. These measurements highlight the structural changes associated with facial aging, which can impact contour and support.

Prior to treatment, dental X‐rays revealed cortical bone thickness of 3.4 ± 0.91 mm on the left side and 3.14 ± 0.49 mm on the right side (Figure 1). These measurements are consistent with age‐related bone loss, particularly in the maxillary and mandibular regions, which are critical for maintaining facial contour and support.

The patient received a series of MWDL treatments targeting the periosteal layer of the maxilla and mandible. The protocol involved the use of multiple wavelengths (755, 808, and 1064 nm, (N.CORE 3D (previous version of Fortra), Classys Inc., Seoul)) to deliver controlled heat stimuli (43°C–46°C) to the periosteum. Each session lasted approximately 30 min, with a total of 16 sessions conducted over 3 months.

**FIGURE 2:**
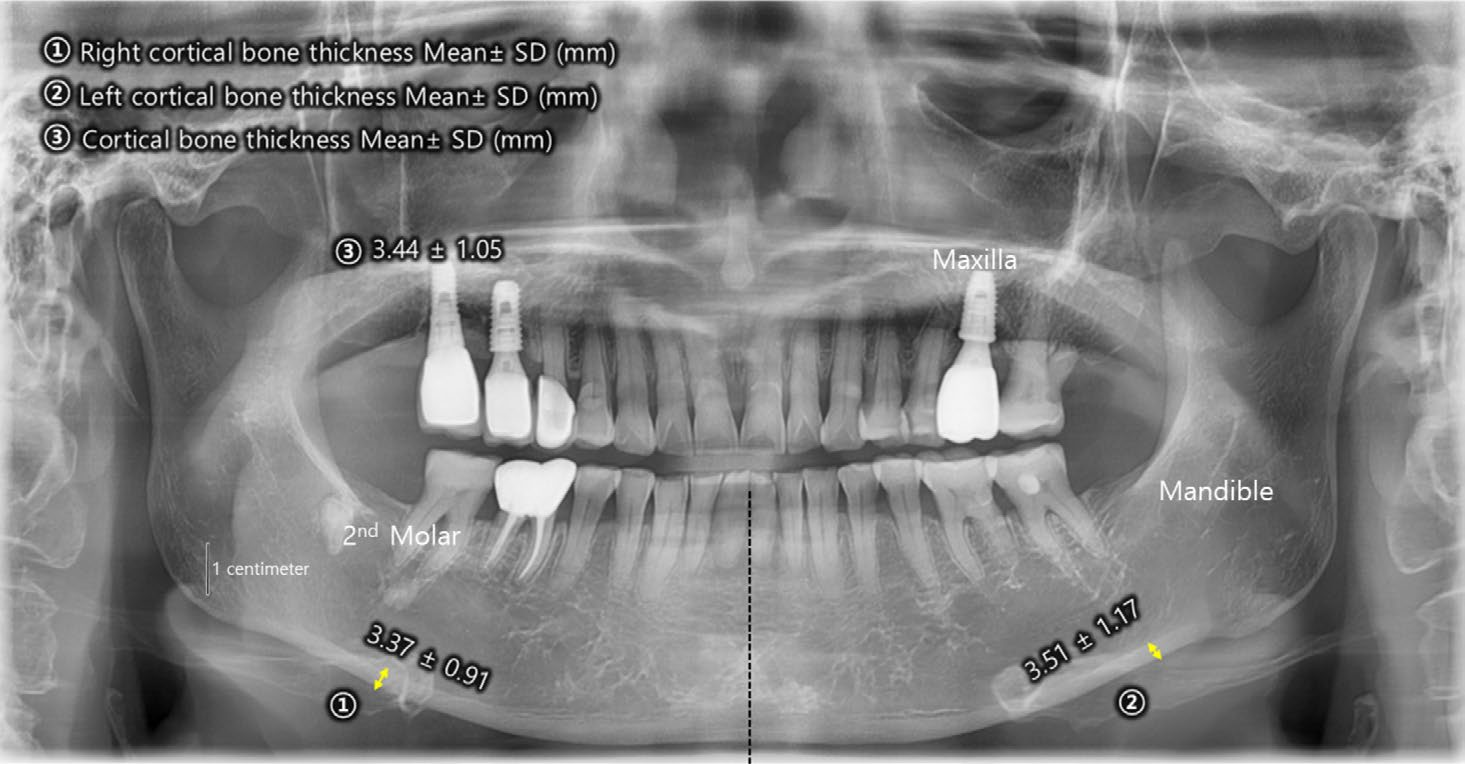
Post‐treatment dental X‐ray taken 3 months after MWDL therapy. Following 16 sessions of MWDL treatment, cortical bone thickness increased to 3.51 ± 1.17 mm on the left side and 3.37 ± 0.91 mm on the right side, suggesting enhanced osteoblast activity and new bone formation. The improvements in bone density demonstrate the potential of MWDL technology in addressing age‐related bony resorption and restoring structural integrity.

Follow‐up dental X‐rays taken 3 months after the final treatment session demonstrated a measurable increase in cortical bone thickness. On the left side, cortical bone thickness increased to 3.51 ± 1.17 mm, while on the right side, it increased to 3.37 ± 0.91 mm (Figure 2). These findings suggest that MWDL treatment successfully stimulated osteoblast activity, leading to new bone formation and improved bone density.

This case study provides preliminary evidence that MWDL technology can effectively promote osteogenesis and address age‐related bony resorption. The observed increase in cortical bone thickness highlights the potential of MWDL to restore structural support in key facial regions, such as the maxilla and mandible. Importantly, these results were achieved non‐invasively, with no reported adverse effects or downtime.

While this case study is promising, it represents a single patient, and further research is needed to validate these findings in larger, more diverse populations. Future studies should explore the long‐term effects of MWDL treatment, optimal treatment parameters, and its efficacy in patients with varying degrees of bone loss.

## 9 Theoretical Basis for Thermally Induced Osteogenesis

Mild thermal stimulation can promote osteogenesis and help prevent bone resorption; however, the effective delivery of external heat into human tissues remains inefficient.

Sun et al. [11] introduces a multifunctional hydrogel that leverages near‐infrared (NIR)‐induced photothermal therapy (PTT) to achieve both outcomes simultaneously. The hydrogel enables controlled, sponge‐like release of osteogenic and antibacterial agents in response to low‐ and high‐intensity NIR light, respectively. Under mild heating, it promotes osteogenesis, while stronger thermal input supports bacterial clearance. In vivo results in rat cranial defects showed nearly a 50% reduction in bone healing time, highlighting the potential of integrated photothermal strategies for efficient bone regeneration with dual therapeutic functions [12].

The study of Tong et al. [13] presents a biodegradable black phosphorus–PLGA osteoimplant that enables efficient mild heat‐induced osteogenesis under NIR irradiation. Even with 7 mm tissue coverage, the implant generates sufficient localized heat to upregulate heat shock proteins and promote bone regeneration. Its degradation yields biocompatible byproducts, including phosphate, supporting bone repair. This NIR‐responsive system offers a promising strategy for non‐invasive, remotely controlled bone defect restoration.

The study of Ye et al. [14] evaluated the synergistic effects of chemical osteogenic stimuli and conditioned media—derived from human fetal osteoblast (hFOB) cells—on the osteogenic differentiation of rabbit bone marrow‐derived mesenchymal stromal cells. The researchers tested conditioned media both with and without prior heat shock treatment (42°C for 1 h) of the hFOB cells, in the presence or absence of standard chemical inducers. Results at days 14 and 21 showed that conditioned media significantly promoted MSC osteogenesis, with the greatest calcium mineralization observed when heat‐shocked conditioned media were combined with chemical inducers. These findings highlight a strong synergistic effect and may help optimize in vitro MSC osteogenesis protocols for regenerative applications.

The study of Zhang et al. [15] prevented early‐stage bone resorption caused by excessive Zn ion release; this study developed a photothermally responsive coating for Zn‐Li alloy implants. The coating stabilized the alloy by minimizing initial corrosion and cytotoxicity, preserving peri‐implant bone integrity. Upon 808‐nm NIR light exposure, controlled degradation was triggered, allowing for safe, gradual Zn ion release and sustained bioactivity. This approach offers a promising strategy to prevent bone resorption while maintaining osseointegration in biodegradable Zn‐based implants.

## 10 Comparison With Other Non‐Invasive Technologies

High‐Intensity Focused Ultrasound (HIFU) and fractional radiofrequency (FRF) are widely used non‐invasive technologies for facial rejuvenation, primarily targeting the dermis and subdermal soft tissues mostly to dermal, subdermal, superficial fatty tissue, and SMAS (superficial muscular aponeurotic system) layers. However, due to the mechanism of delivering the energy, they are not able to push the energy deep into the soft tissues; these modalities are limited in their ability to reach the periosteal layer or directly influence the underlying bone. Today, HIFU comes out with deeper layer targeted below the SMAS layer with 9, and 13 mm (Ultraformer MPT, Classys Inc., Seoul), and may reach the periosteal layer; however, exactly targeting the heat to the periosteal layer would be difficult since the face has uneven depth of the soft tissue. In contrast, MWDL is capable of delivering bulky thermal stimuli deep to the periosteum, stimulating osteoblast activity and promoting osteogenesis.

## 11 Safety Considerations and Future Research

As with any heat‐based therapy, patient safety remains a critical consideration. Proper calibration of the diode laser's energy settings is essential to avoid complications, and individual factors such as bone density and health status must be considered. Future research should focus on refining treatment protocols and exploring the long‐term effects of MWDL on facial bone regeneration. Comparative studies with other bone‐regenerative therapies will also be valuable in understanding where MWDL fits within the broader landscape of aesthetic treatments.

This clinical commentary has several important limitations. First, the findings are based on a single patient case study, resulting in a very small sample size that limits the generalizability of the observations. Second, there was no control group for comparison, which precludes definitive conclusions regarding the causative effects of MWDL treatment. Third, the follow‐up period was relatively short, with imaging conducted 3 months after the final session, making it difficult to assess the long‐term stability of the observed bone changes. Lastly, the hypothesis that MWDL can stimulate periosteal osteogenesis remains speculative at this stage and requires validation through larger, controlled clinical studies and long‐term observational data. These limitations must be carefully considered when interpreting the preliminary results presented in this commentary.

## 12 Conclusion

Bony resorption plays a significant yet often neglected role in the aging process. By directly stimulating osteoblast activity and promoting bone regeneration, MWDL offers a promising solution for addressing bone loss in facial rejuvenation. With its ability to target the periosteal layer precisely, MWDL has the potential to provide long‐lasting improvements in facial structure and contour, moving beyond the limitations of soft tissue‐focused treatments.

As research progresses, MWDL technology could redefine approaches to facial aging by addressing skeletal changes rather than merely masking them with temporary interventions. Its integration into clinical practice may offer patients a more comprehensive and effective solution for achieving a youthful and structurally sound facial appearance.

## Author Contributions

All authors have reviewed and approved the article for submission. Conceptualization: Jovian Wan, Song Eun Yoon, Jong Keun Song, Isaac Kai Jie Wong, Tingsong Lim, Jin‐Hyun Kim, Soobin Kim, and Kyu‐Ho Yi. Writing – original draft preparation: Jovian Wan. Writing – review and editing: Jovian Wan, Song Eun Yoon, Jong Keun Song, Tingsong Lim, Jin‐Hyun Kim, Soobin Kim, and Kyu‐Ho Yi. Supervision: Kyu‐Ho Yi.

## Conflicts of Interest

The authors declare no conflicts of interest.

## Data Availability

The authors have nothing to report.
